# Understanding the Liquid States of Cyclic Hydrocarbons Containing N, O, and S Atoms via the 3D-RISM-KH Molecular Solvation Theory

**DOI:** 10.3390/molecules27196563

**Published:** 2022-10-04

**Authors:** Dipankar Roy, Andriy Kovalenko

**Affiliations:** 1 Department of Biological Sciences, University of Alberta, Edmonton, AB T6G 2E9, Canada; 2 Nanotechnology Research Centre, National Research Council of Canada, 11421 Saskatchewan Drive, Edmonton, AB T6G 2M9, Canada

**Keywords:** heterocyclic compounds, molecular solvation theory, 3D-RISM-KH, force field, partial atomic charges, solvation free energy

## Abstract

The 3D-reference interaction site model (3D-RISM) molecular solvation theory in combination with the Kovalenko–Hirata (KH) closure is extended to seven heterocyclic liquids to understand their liquid states and to test the performance of the theory in solvation free energy (SFE) calculations of solutes in select solvents. The computed solvent site distribution profiles were compared with the all-atom molecular dynamics (MD) simulations, showing comparable performances. The computational results were compared against the structural parameters for liquids, whenever available, as well as against the experimental SFEs. The liquids are found to have local ordered structures held together via weak interactions in both the RISM and MD simulations. The 3D-RISM-KH computed SFEs are in good agreement with the benchmark values for the tetrahydrothiophene-S,S-dioxide, and showed comparatively larger deviations in the case of the SFEs in the tetrahydrofuran continuum.

## 1. Introduction

Molecular solvation is essential to life processes. While a qualitative description of the solvation process is reported in numerous reports, quantitative applications are small in number, in comparison to the former. The difficulty is in the theoretical formulations of how solvents behave around a solute, providing a dielectric medium via polarity, as well as interacting directly with the solute(s) [1,2,3,4,5]. Explicit solvation models capture the dielectric effect of the solvent around a solute and showed to be effective in predicting and explaining chemical (re)activity [4,5,6,7,8]. The quantum chemical solvation models based on atomic surface charge models, i.e., COSMO, PCM, and SMD are a good example of this approach [7,8,9,10,11,12]. The inclusion of explicit solvent molecules, in combination with a continuum solvation model, also known as the cluster–continuum model, was used to account for explicit interactions between solvent and solute molecules [13,14,15,16]. Such models are very useful but limited to smaller sizes due to the large computational cost associated with electronic structure calculations. The molecular dynamic simulations use explicit solvent molecules defined via standard force field parameters to account for solute–solvent interactions. The statistical mechanics-based molecular solvation theory via the reference interaction site model (RISM, 1D, and 3D) is another prominent solvation model with vast applications in chemical, biological, and material science applications. The RISM formalism is rooted in the works of Chandler, Anderson, and coworkers [17,18,19,20,21,22]. The RISM provides probability density *ρ_γ_g_γ_*(*r*) of finding interaction site γ of solvent molecules at position *r* around a solute molecule of arbitrary shape. The *ρ_γ_* and *g_γ_* are average number density in the bulk and normalized density distribution, respectively. The 3D-total correlation function, *h_γ_*(***r***), is connected to the 3D-direct correlation function, *c_α_*(***r***), via the site–site bulk solvent susceptibility *χ_αγ_*(*r*), for solvent sites *α* around the solute as:

$$h_{\gamma }\left(r\right)=\underset{\alpha }{\sum }\int dr^{\prime }c_{\alpha }\left(r-r^{\prime }\right)\chi_{\alpha \gamma }\left(r^{\prime }\right)$$

The susceptibility *χ_αγ_* is obtained from the site density and intramolecular correlation function, *ω_αγ_*(*r*), from the dielectrically consistent RISM (DRISM) equation [23]: *χ_αγ_*(*r*) = *ω_αγ_*(*r*) + *ω_αγ_*(*r*) *ρ_γ_h_αγ_*(*r*).

A closure relation is required for integrating an infinite chain of interactions involving intra- and intermolecular sites in order to get the distribution functions. The closure form proposed by Kovalenko and Hirata (KH) has the following functional form [24]:

$$g_{\gamma }\left(r\right)=\{\begin{array}{ccc} \exp\left(-u_{\gamma }\left(r\right)/\left(k_{B}T\right)+h_{\gamma }\left(r\right)-c_{\gamma }\left(r\right)\right) & \mathrm{for} & g_{\gamma }\left(r\right)\leq 1\\ 1-u_{\gamma }\left(r\right)/\left(k_{B}T\right)+h_{\gamma }\left(r\right)-c_{\gamma }\left(r\right) & \mathrm{for} & g_{\gamma }\left(r\right)>1 \end{array}$$

The KH closure is known for providing numerical stability and easier convergence in computing 3D distribution functions for liquids of different polarities. The excess chemical potential (*μ*) is obtained from a close analytical form:

$$\mu_{\mathrm{solv}}=\underset{\gamma }{\sum }\int_{V}dr\;\Phi_{\gamma }\left(r\right)$$

and

$$\Phi_{\gamma }\left(r\right)=\rho_{\gamma }k_{B}T\left[\frac{1}{2}h_{\gamma }^{2}\left(r\right)\Theta \left(-h_{\gamma }\left(r\right)\right)-c_{\gamma }\left(r\right)-\frac{1}{2}h_{\gamma }\left(r\right)c_{\gamma }\left(r\right)\right]$$

where Θ(*x*) is the Heaviside step function. The solvation free energy functional is written in the 3D-RISM-KH formalism via Gaussian fluctuation (GF) free energy:

$$\Delta G_{\mathrm{solv}}^{\mathrm{GF}}=k_{B}T\underset{\gamma }{\sum }\rho_{\gamma }\int_{R^{3}}[-c_{\gamma }\left(r\right)-\frac{1}{2}c_{\gamma }\left(r\right)h_{\gamma }\left(r\right)]\;dr$$

Interested readers are referred to Refs. [18,25,26,27] for the detailed theoretical derivation of the RISM theory and correlation functions mentioned here. The errors in 3D-RISM computations are results of erroneous calculation of internal pressure as well as inadequacies of the force field parameters used. Several correction schemes were developed to scale RISM-computed Δ*G^GF^* to correctly correlate with solvation free energy. The most used “universal correction” scheme uses a linear regression scheme using RISM-computed partial molar volume (PMV) for scaling and has the following form [25]: Δ*G*_corrected_ = Δ*G*^GF^ + *a* ∙ PMV + *b*, and the parameters *a* and *b* are obtained from regression analysis.

**Figure 1:**
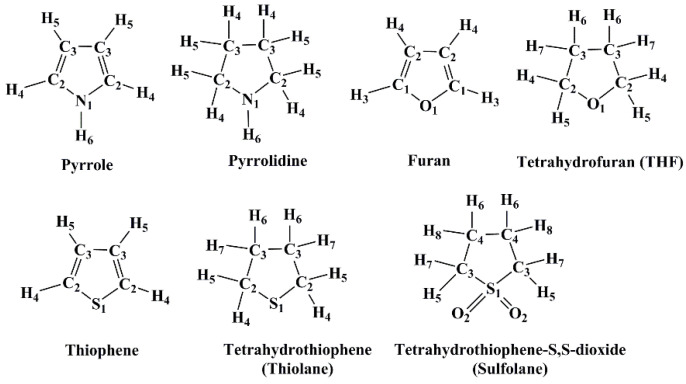
Cyclic molecules studied using the 3D-RISM-KH molecular solvation theory in this work. Equivalent solvent sites are marked with numbers. The highest number refers to the number of solvent sites present in each molecule.

The 3D-RISM-KH has been used successfully to model solvents of varying polarities, e.g., water, hydrocarbons, octanol, amines, ketones, etc., to name a few. For a review of the applications of the RISM-KH theory, please see Refs. [26,27,28]. The benchmarking of this theory to a new set of solvents depends on the quality of the data available in the literature. For example, diffraction data on the liquid form are the most suitable to judge the accuracy of RISM calculations. Solvation free energy (SFE) of solutes provides an additional test for the RISM models. However, these benchmark data are not often available in the literature. In the present work, we tested the performance of the 3D-RISM-KH molecular solvation theory in modeling the liquid state of seven heterocyclic ring systems containing N, O, and/or S atoms. The molecules we chose were: Pyrrole, Pyrrolidine, Furan, Tetrahydrofuran (THF), Thiophene, Tetrahydothiophene (thiolane), and Tetrahydrothiophene-S,S-dioxide (sulfolane) (Figure 1). The modeling of ring structures, especially the ones containing heteroatoms is often problematic due to inadequate force field parameters. We tested the well-used generalized AMBER force field (GAFF) and the universal force field (UFF) parameters in this study [29,30,31]. Additionally, we have compared the 3D-RISM-KH computed SFEs of 14 solutes in THF and sulfolane solvents against those reported in the MNSol database [32]. The 3D-RISM-KH computed solvent profiles are compared against those computed using the molecular dynamics (MD) simulations, as well as against experimental data, whenever available. The present work is part of an ongoing effort to develop reliable solvent models for applications using the 3D-RISM molecular solvation theory.

## 2. Results

In this work, we compared and contrasted the 3D-RISM-KH and MD computed liquid state structures of seven heterocyclic liquids against available experimental data. For the RISM calculations, GAFF and UFF force field parameters were used. We experienced convergence issues for the solvent susceptibility calculations of the pure unsaturated rings (pyrrolidine, tetrahydrothiophene, tetrahydrofuran, and sulfolane) using the GAFF force field parameters with AM1-BCC charges. Such issues were not noticed for the aromatic rings. The UFF parameters in combination with the MP2/cc-pVTZ level computed atomic charges worked well for all seven systems. In this manuscript, all the reported pure liquid pairwise distribution functions (PDFs) are computed from the UFF-parameter-based solvent models. For the GAFF-computed parameters of aromatic heterocycles, please refer to the Supplementary Materials (Figures S1–S3). We observed noticeable differences between the GAFF- and UFF-computed distributions for the three liquid aromatic heterocycles. Further work is underway to compare the effect of force field parameters in aromatic systems via the 3D-RISM-KH theory. The GAFF parameters used for the all-atom MD simulation resulted in equilibrated systems. The physical parameters of the seven liquids and those obtained from the MD simulations are provided in Table 1. The structures of the individual liquids are discussed in the following sections. The solvation free energy (SFE) data in the two liquids are provided in the final section.

### 2.1. Liquid Structure of Pyrrole

**Figure 2:**
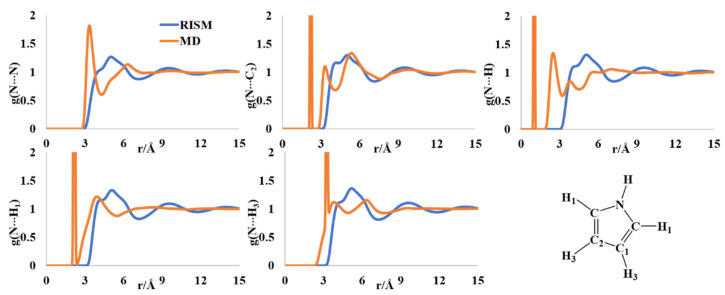
RISM- and MD-computed distribution functions for liquid pyrrole.

The aromatic pyrrole molecule is an important synthetic block in organic and medicinal chemistry. The molecule is known to form clusters from gas phase and solid phase studies [36,37,38]. The experimental results of the liquid structure of pyrrole are sparse [39,40]. The liquid clusters were reported to have [N]-H···π interactions forming T-shaped dimers from the molecular simulations as well as vibrational spectroscopy [41,42]. The N-N radial distribution function (rdf) showed multiple minima in the MD and RISM computations. The MD rdf has the first peak at ~3.4 Å and the second broad one at ~6.4 Å (Figure 2). The RISM-computed PDF has a broad peak with a shoulder in the region ~3.8–5.2 Å. The multiple peaks in the MD profile were reported previously [43,44]. The rdf of N-C_2_ has one sharp peak at 2.2 Å and two nearby peaks at 3.4 Å and 5.5 Å. The first one is the intramolecular separation, which is not calculated in the RISM formalism. The RISM-computed PDFs for the same have a broad peak with multiple shoulders in the rebroad region of 3.9–6.2 Å. The N-H intermolecular distribution has maxima at 2.6 and 3.8 Å, and a broad region between ~5–7 Å. These findings are similar to those reported by Barone and coworkers [44] and were indicative of T-shaped H-π interactions. The RISM-computed PDFs for all the N-H intermolecular distributions have multiple peaks with a multishoulder broad peak between ~3–7 Å, indicative of the existence of different clusters in the liquid form.

### 2.2. Liquid Structure of Furan

Similar to pyrrole, experimental data on the structure of furan are not present in the literature, except for the solid phase and energy dispersive X-ray diffraction of the liquid [40,45]. The latter, however, provided structural factors processed with a sharpening factor, making it impossible to make a direct comparison. The authors in that work [45] pointed to the presence of the C-H···π, C-H···O, and H···H interaction in a complex long-range structure of the liquid.

**Figure 3:**
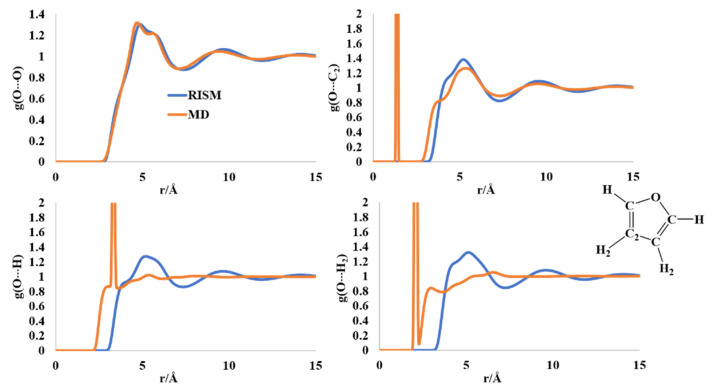
RISM- and MD-computed distribution functions for liquid furan.

The O-O and O-C_2_ intermolecular distributions computed by the MD and RISM formalisms are comparable (Figure 3). For the O-O distribution function, the first peak with a shoulder appears at 4.6–5.8 Å region, followed by a broad peak at ~10 Å. The O-C_2_ distribution has the first broad maxima in the region of 3.7–5.5 Å, for both the MD and RISM calculations, pointing to probable π-stacking interactions. The O-H (and H_2_) distribution functions differ in shape between MD and RISM. The MD computed RDF for O-H intermolecular distribution has a sharp peak at 3.42 Å. This peak is replaced in the RISM-computed PDF by a broad peak in the region ~4–6.5 Å. This broad region is present in the PDF of the O-H_2_ intermolecular distribution. The RISM computations are indicative of weak O···H(H_2_) interactions in the liquid state that could contribute to ordered structure.

### 2.3. Liquid Structure of Thiophene

**Figure 4:**
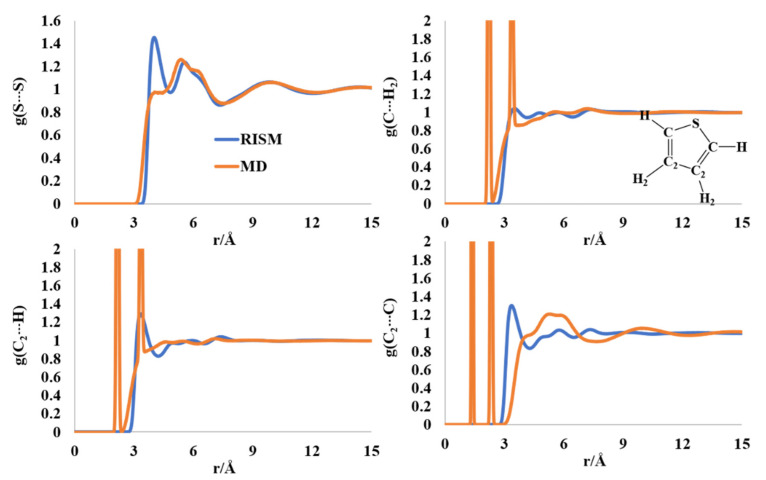
RISM- and MD-computed distribution functions for liquid thiophene.

The S-S intermolecular PDF has peaks around 4.1 and 5.8 Å for thiophene. The MD computed rdf has a shoulder around 4 Å and a shouldered peak at ~5.5–6.3 Å (Figure 4). The two sharp peaks at ~2.1 Å and ~3.3 Å in the C(C_2_)-H_2_(H) rdfs are intramolecular in nature, much as the two sharp peaks in the C-C2 rdf at 1.32 Å and ~2.3 Å. The C-H PDFs and rdfs did not provide sufficient information on the orderliness in the liquid thiophene structure. The first peak of the C-C_2_ PDF was found at 3.5 Å, followed by a multishoulder hump in the region of 4.5–8 Å, which is present in the MD profile, too. The orderliness in the furan molecule seemed to be less than that of those in furran and pyrrole, although some ordered structures cannot be ruled out from the rdfs and PDFs, as reported in previous works [40,45].

### 2.4. Liquid Structure of Pyrrolidine

**Figure 5:**
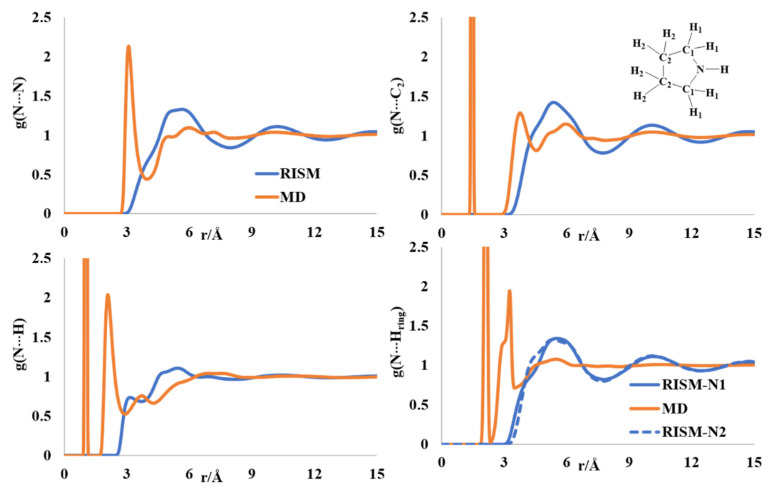
RISM- and MD-computed distribution functions for liquid pyrrolidine.

The ring puckering of pyrrolidine received a lot of attention, and gas phase diffraction and theoretical modeling reports are present on the puckered ring of pyrrolidine [46,47,48,49], although complete structural descriptions of the liquid state are absent in the chemical literature. The N-N rdf has a sharp peak at 3 Å and a broad peak in the region of 4.7–8 Å (Figure 5). The sharp peak is absent in the RISM PDF, though the broad peak is present in the aforementioned region. The N-C2 intermolecular distribution showed multiple minima in the region of 3.8–6 Å, with a broad region after 9 Å in both the MD and RISM computations. The intermolecular N-H distribution has two maxima at 2.8 and 3.9 Å in the MD profile. The first maximum in the RISM PDF appeared at ~3.4 Å. The position of the maximum in the intermolecular N-H(ring) distributions is comparable between the RISM and MD profiles. The presence of at least two dimeric forms was reported from spectroscopic analysis [50]. The intermolecular atomic distribution profiles computed by MD and RISM support this observation.

### 2.5. Liquid Structure of Tetrahydrofuran (THF)

**Figure 6:**
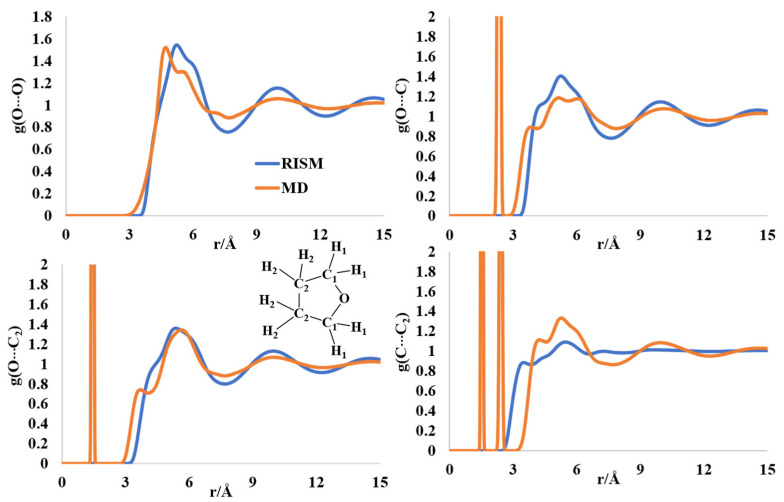
The RISM- and MD-computed distribution functions for liquid THF.

The structure of THF has received attention due to a flexible ring structure, like pyrrolidine, and discrepancies between the computed and experimental structure parameters in the liquid form [51,52,53,54]. The first maxima (two peaks) in the intermolecular O-O distribution are found at 4.6 Å (5.6 Å) and 5.31 Å (6.0 Å), for the MD and RISM calculations, respectively (Figure 6). The experimentally determined maximum for this distribution was reported at 5.3 Å (6.3 Å) [53]. The O-C intermolecular distribution has multipeak broad maxima in the region of ~4–6.4 Å from MD and RISM. The experimental distributions had two peaks at 4.2 Å and 6.8 Å. The O-C2 intermolecular peaks are at 3.8 Å and 6 Å for MD, while RISM had a broad peak with a shoulder at 5.6 Å. The experimentally reported maxima were at 4.2 Å and 7.9 Å [53]. Finally, the C-C2 intermolecular distribution peaks were obtained at 4.3 Å and 5.5 Å for the MD simulations, and at 3.4 Å and 5.5 Å for the RISM computations. The experimental maxima for this intermolecular separation were at 4.4 Å and 7.4 Å. The differences between the experimental rdfs and the theoretically computed ones were reported previously [54,55].

### 2.6. Liquid Structure of Tetrhydrothiophene (Thiolane)

**Figure 7:**
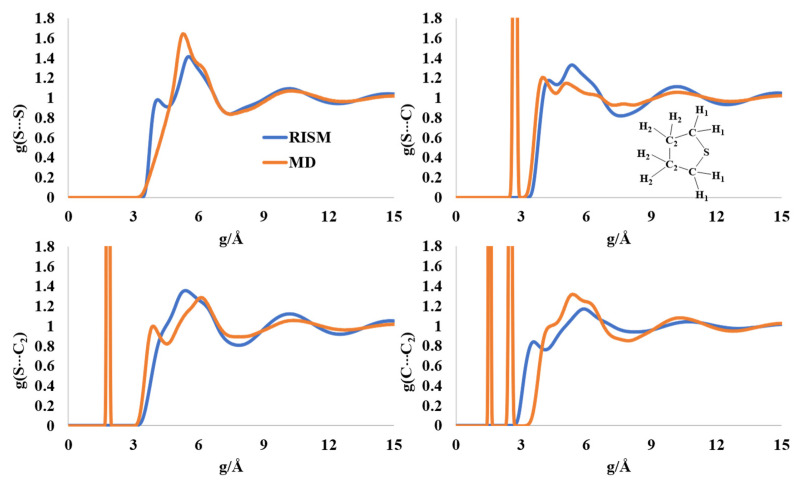
RISM- and MD-computed distribution functions for liquid thiolane.

The RISM- and MD-computed intermolecular distribution profiles have a qualitative similar structure for liquid thiolane, although the positions of the maxima varied. The intermolecular S-S distribution has maxima at 4.2 Å and 5.7 Å in the RISM profile, and at 5.4 Å for the MD simulations (Figure 7). These peak positions are longer than the experimentally reported one at 2.65 Å [56]. The multiple maxima in the broad peak in the region of 3.9 Å-7 Å for the S-C(C_2_) intermolecular distribution can be interpreted as possible ordered structures in the liquid form. The C-C_2_ intermolecular distribution profile also points to possible cluster form(s).

### 2.7. Liquid Structure of Tetrahydrothiophene-S,S-dioxide

**Figure 8:**
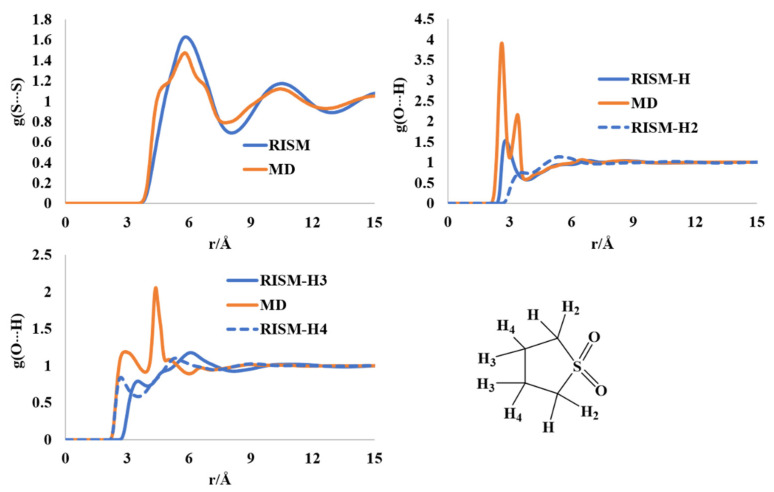
RISM- and MD-computed distribution functions for liquid sulfolane.

The sulfolane molecule (and the class of compounds) have extensive applications in the field of electrochemistry and battery technology. The planarity of the ring and puckerings were investigated using diffraction and spectrochemical analysis in the solid and liquid phases [57,58,59,60,61,62]. Despite these reports, a full description of the liquid state as well as unambiguous ring puckering is absent in the literature. The interatomic S-S distribution has maxima at ~6 Å and 10.5 Å in both the RISM and MD profiles. The atomic charges computed at the MP2/cc-pVTZ level for the RISM calculations reflected the electronic distribution differently than the AM1-BCC charges, especially for H-atoms. The AM1-BCC charges have two distinct sets of hydrogens, those attached to the carbon next to the S atom and those connected to the carbon atom(s) farthest from the S atom. The MP2/cc-pVTZ level charges divided the hydrogens into four different groups based on the envelope ring structure, where C-atoms adjacent to S are connected to two different types of hydrogens, and the two C-atoms away from S are also attached to two different types of H-atoms, solely based on atomic charges (Figure 8). The first two maxima in the intermolecular O···H(H_2_) distribution are found at 2.64 Å (2.9 Å in RISM) and 3.44 Å (3.5 Å in RISM) via the MD simulation. These findings are in qualitative agreement with the previous report on forcefield development for the sulfolane molecule [62]. Overall, the liquid sulfolane molecules are held together by O···H[C] weak hydrogen bonds.

### 2.8. SFEs in Tetrahydrofuran (THF) and Tetrahydrothiophene-S,S-dioxide (Thiolane) Solvents

The fourteen solutes (seven for each solvent) for which experimental solvation free energies were available in THF and sulfolane medium were computed using the 3D-RISM-KH theory. The solutes are parameterized using the UFF parameters with AM1 charges. The Gaussian fluctuation excess chemical potentials computed via the 3D-RISM-KH theory were corrected via the “universal correction” scheme. The fitting coefficients are provided in Table 2.

The corrected SFEs are provided in Table 3. The small number of experimental data is indeed a handicap in the calibration process. The computed SFEs in THF have a mean unsigned error (MAE) of 4.3 kcal/mol) and a relative mean squared error (RMSE) of 6.2 kcal/mol. The MAE and RMSE for the solvation free energies in sulfolane are 0.63 and 0.92 kcal/mol, respectively. The large RMSE values in the THF solvent point to further improvements required in the force field parameters for the solvent molecule, as well as a need for a larger benchmark dataset.

## 3. Discussion

In this manuscript, we have explored the liquid states of seven heterocycles (aromatic and anon-aromatic) via molecular simulations. The 3D-RISM-KH theory and MD simulations are used for these all-atom simulations of the liquid state. The intermolecular radial distribution functions obtained from the MD simulations are qualitatively similar to the distributions computed via the RISM formalism, although some discrepancies are noted in the intermolecular distribution function involving hydrogen atoms. It is not possible to compare the accuracy of the two theoretical models, as there is not sufficient experimental data available to compare and contrast. However, one can summarize the local molecular cluster in these liquids held together by H···π, H···X(X=N/O) interactions for N- and O-containing systems. While the clustering in the thiophene and tetrahydrothiophene is less prominent than in the other liquids, one cannot rule out the effect of the dispersion effects, which are not treated explicitly in either of the approaches.

The 3D-RISM-KH computations of the SFEs in the two solvents provided encouraging results. The THF solvation model requires further improvements for use in the SFE applications, though the distribution profiles are in general in agreement with experimental findings.

The differences between the MD and RISM calculations of the solvent site distributions have some contributions from the differences in the force field parameters employed. Development of force field parameters for saturated ring systems is a non-trivial task. We have first-hand experience of difficulties in obtaining physically meaningful solvent susceptibility functions using available force field parameters for cyclic molecules [63,64]. The performances of the UFF parameters with the MP2/cc-pVTZ level atomic charges for RISM-KH provided reasonable results, in comparison to available experimental results. We noticed that the GAFF-computed RISM distributions have peak positions different than those from the UFF parameters. The PDFs for pyrrole from the RISM calculations are an interesting observation, as they have similar distribution profiles for all heavy atoms. The results may simply be that the force field in combination with RISM did not work for pyrrole. The N-containing heterocycles have been known to have issues with the GAFF parameters [65].

In this manuscript, solvent models of seven heterocyclic compounds were developed within the framework of the 3D-RISM-KH theory. These solvent models provided good structural and physical features when compared with the molecular dynamics simulations and available experimental results. These solvent models can be used for further applications in (bio)chemical research, and also as fragments for binding site mapping on biological receptor surfaces, using the molecular solvation theory.

## 4. Materials and Methods

The RISM-KH calculations were completed using our in-house code, a working version of which is implemented in the AMBERTOOLS software package [66]. The RISM-KH calculations were performed using the all-atom GAFF and UFF force field parameters. The former is combined with the AM1-BCC charges [29,30]. The UFF force field is combined with the atomic charges computed at the MP2/cc-pVTZ level on the B3LYP/cc-pVTZ level optimized geometries [31,66,67,68,69,70,71]. The electronic structure calculations were performed using the Gaussian16 suite of quantum chemical software [72]. For solvent susceptibility calculations, the extended-RISM (X-RISM) formalism was employed. The modified-DIIS convergence criterion (MDIIS) is set to 1E-10 for all the calculations. The reliability converged solvent susceptibility functions were verified using computed compressibility of the liquid. The equivalent solvent sites for pure liquids are numbered in Figure 1.

The benchmark solvation free energies in THF and sulfolane were obtained from the Minnesota solvation database (MNSol 2009) and consist of 14 datapoints [32]. For the 3D-RISM-KH calculations, we have used the geometry obtained from this database. The 3D-RISM-KH computed Gaussian fluctuation excess chemical potentials are corrected using multiple linear regressions. We have used the UFF force field parameters with semiempirical AM1 atomic charges for solute molecules [73]. The 3D-RISM-KH calculations on the solutes were performed on a uniform cubic 3D grid of 128 × 128 × 128 points in a box of size 64 × 64 × 64 Å^3^. For a sample workflow for a RISM-KH-based molecular simulation, please see reference [27].

All the molecular dynamics (MD) simulations were performed using the GROMACS-2020.4 engine with the GAFF force field parameters and AM1-BCC charges for all the molecules in the liquid state [74]. A cubic simulation box consisting of 256 molecules was used for each system without any restraints. Energy minimized systems were subjected to 20 ns of NVT and NPT equilibration. The temperature and density profiles were checked from the resultant simulations to confirm equilibrated systems for further production simulations of 50 ns. All the trajectory analyses were performed using the modules provided in the GROMACS software package.

## Figures and Tables

**Table 1:** Density (standard, and MD-computed) and dielectric constants used in this work for seven liquids.

Molecule	Dielectric Constant	Density (Experimental, g·cm^−3^)	Density (MD, g·cm^−3^) ^1^
Pyrrole	8.00	0.968 [33]	1.009
Furan	2.94	0.973 [34]	0.964
Thiophene	2.73	1.065 [33]	1.033
Pyrrolidine	8.30	0.852 [34]	0.892
Tetrahydrofuran (THF)	7.42	0.883 [33]	0.899
Tetrahydrothiophene	8.61	0.998 [35]	0.977
Sulfolane	43.96	1.2606 [34]	1.298

^1^ Computed using the GAFF force field with AM1-BCC charges.

**Table 2:** Regression coefficients (a and b) used for the universal correction fitting of the solvation free energies obtained via the 3D-RISM-KH theory.

Solvent	a (kcal/mol/Å^3^)	b (kcal/mol)
THF	−0.1139	−10.5505
Sulfolane	−0.1715	−7.1919

**Table 3:** Experimental SFEs (Δ*G*_expt_), 3D-RISM-KH computed excess chemical potential (*μ*^GF^), partial molar volume (PMV), and corrected SFEs (Δ*G*_cor_).

Solute	Solvent	Δ*G*_expt_ (kcal/mol)	*μ*^GF^ (kcal/mol)	PMV (Å^3^)	Δ*G*_cor_ (kcal/mol)
n-octane	THF	−5.39	44.13	352.55	−6.57
toluene	THF	−5.5	28.46	235.24	−8.88
ethanol	THF	−4.56	21.21	154.02	−6.88
tetrahydrofuran	THF	−4.25	24.21	192.77	−8.29
1,4-dioxane	THF	−5.17	44.06	207.79	9.84
2-butanone	THF	−4.54	27.72	212.46	−7.03
nitromethane	THF	−5.09	21.21	152.51	−6.71
n-octane	sulfolane	−2.44	53.41	281.17	−2.02
toluene	sulfolane	−4.23	33.32	188.49	−6.21
ethanol	sulfolane	−4.3	22.90	114.90	−4.00
1,4-dioxane	sulfolane	−4.9	30.16	163.54	−5.09
2-butanone	sulfolane	−4.09	31.08	163.25	−4.12
butylamine	sulfolane	−4.25	35.56	182.73	−2.98
nitromethane	sulfolane	−5.28	20.98	110.01	−5.09

## Data Availability

All pertinent data related to this paper are provided in the Supplementary Materials.

## Supplementary Materials

The following are available online at https://www.mdpi.com/article/10.3390/molecules27196563/s1, **Table S1.** B3LYP/cc-pVTZ level optimized geometries of seven heterocycles studied: Pyrrole, Pyrrolidine, Furan, Tetrahydrofuran, Thiophene, Tetrahydrothiophene, Tetrahydrothiophene-S,S-dioxide, **Figure S1.** GAFF and UFF force field computed pairwise distribution functions of liquid pyrrole via the 3D-RISM-KH theory, Figure S2. GAFF and UFF force field computed pairwise distribution functions of liquid furan via the 3D-RISM-KH theory, **Figure S3.** GAFF and UFF force field computed pairwise distribution functions of liquid thiophene via the 3D-RISM-KH theory.
