# Supplementary material for: Understanding the Liquid States of Cyclic Hydrocarbons Containing N, O, and S Atoms via the 3D-RISM-KH Molecular Solvation Theory
Source: Molecules. 2022 Oct 4;27(19):6563. doi: 10.3390/molecules27196563 (PMC9572648; doi:10.3390/molecules27196563)
Supplement: Supplementary file 1 [file molecules-27-06563-s001.zip › supplrmentary.pdf]

Supplementary Materials

for

Understanding the liquid states of cyclic hydrocarbons containing N-, O-, and S-atom via the 3D-RISM-KH molecular solvation theory

Dipankar Roy[1] and Andriy Kovalenko[1,2*]

[1]Department of Biological Sciences, University of Alberta, Edmonton, Alberta T6G 2E9, Canada

[2]Nanotechnology Research Centre, National Research Council of Canada, 11421 Saskatchewan Drive, Edmonton, Alberta T6G 2M9, Canada

[*]Correspondence: andriy.kovalenko@ualberta.ca

Table S1. B3LYP/cc-pVTZ level optimized geometries of seven heterocycles studied:

Pyrrole

Standard orientation:

Center Number	Atomic Number	Atomic Type	Coordinates (Angstroms) X	Y	Z
1	7	0	1.118499	-0.000043	-0.000098
2	6	0	0.330504	-1.121651	0.000156
3	6	0	0.330590	1.121627	0.000063
4	6	0	-0.979854	-0.710496	-0.000156
5	6	0	-0.979798	0.710570	0.000054
6	1	0	2.121791	-0.000084	-0.000164
7	1	0	0.761370	-2.107247	0.000253
8	1	0	0.761533	2.107189	0.000103
9	1	0	-1.841469	-1.355894	-0.000288
10	1	0	-1.841364	1.356035	0.000081

Pyrrolidine

Standard orientation:

Center Number	Atomic Number	Atomic Type	Coordinates (Angstroms) X	Y	Z
1	7	0	0.003519	-1.169810	-0.349005
2	6	0	-0.780634	1.023297	-0.058199
3	6	0	0.773052	1.029439	-0.054388
4	6	0	-1.160575	-0.449809	0.170700
5	6	0	1.164332	-0.442240	0.167843

6	1	0	-1.201506	1.669262	0.711011
7	1	0	-1.160015	1.367287	-1.019022
8	1	0	1.154830	1.383697	-1.010496
9	1	0	1.184462	1.673348	0.721637
10	1	0	-1.324820	-0.629287	1.244934
11	1	0	-2.067159	-0.743683	-0.359052
12	1	0	2.070753	-0.727753	-0.366730
13	1	0	1.334628	-0.623992	1.240669
14	1	0	0.007143	-2.144336	-0.075647

Furan

Standard orientation:

Center Number	Atomic Number	Atomic Type	Coordinates (Angstroms) X	Y	Z
1	8	0	-0.000048	-1.157231	-0.000076
2	6	0	0.715942	0.955504	-0.000023
3	6	0	-0.715869	0.955556	0.000071
4	6	0	1.092595	-0.345706	0.000053
5	6	0	-1.092615	-0.345628	-0.000018
6	1	0	1.370382	1.809647	-0.000045
7	1	0	-1.370244	1.809746	0.000122
8	1	0	2.046785	-0.840032	0.000074
9	1	0	-2.046852	-0.839869	-0.000047

Tetrahydrofuran

Standard orientation:

Center Number	Atomic Number	Atomic Type	Coordinates (Angstroms) X	Y	Z
1	8	0	-0.000000	-1.249343	-0.000004
2	6	0	-0.733337	0.993926	-0.223125
3	6	0	0.733336	0.993927	0.223122
4	6	0	-1.166422	-0.428829	0.129855
5	6	0	1.166423	-0.428829	-0.129850
6	1	0	-1.337045	1.752497	0.273367
7	1	0	-0.801821	1.157705	-1.300168
8	1	0	1.337044	1.752497	-0.273372
9	1	0	0.801820	1.157714	1.300164
10	1	0	-1.535550	-0.485232	1.160198
11	1	0	-1.942489	-0.818188	-0.531054
12	1	0	1.535562	-0.485235	-1.160188
13	1	0	1.942483	-0.818187	0.531068

Thiophene

Standard orientation:

Center Number	Atomic Number	Atomic Type	Coordinates (Angstroms) X	Y	Z
1	16	0	1.192763	-0.000001	-0.000343
2	6	0	-1.266968	-0.711536	0.000060
3	6	0	-1.266965	0.711539	0.000024
4	6	0	-0.009732	-1.238025	0.000285
5	6	0	-0.009730	1.238025	0.000373

6	1	0	-2.162947	-1.314326	0.000051
7	1	0	-2.162946	1.314328	0.000002
8	1	0	0.281025	-2.274571	0.000413
9	1	0	0.281031	2.274569	0.000568

Tetrahydrothiophene

Standard orientation:

Center Number	Atomic Number	Atomic Type	Coordinates (Angstroms) X	Y	Z
1	16	0	-1.313657	0.000007	-0.000009
2	6	0	1.282944	0.715436	0.270341
3	6	0	1.282923	-0.715460	-0.270360
4	6	0	-0.051074	1.342144	-0.135435
5	6	0	-0.051096	-1.342133	0.135468
6	1	0	1.364172	0.696710	1.359302
7	1	0	2.122209	1.297977	-0.113689
8	1	0	1.364104	-0.696736	-1.359324
9	1	0	2.122187	-1.298019	0.113640
10	1	0	-0.342528	2.165517	0.512810
11	1	0	-0.025619	1.701670	-1.163387
12	1	0	-0.342576	-2.165534	-0.512731
13	1	0	-0.025621	-1.701613	1.163437

Tetrahydrothiophene-S,S-dioxide

Standard orientation:

Center Number	Atomic Number	Atomic Type	Coordinates (Angstroms) X	Y	Z
1	16	0	-0.785928	-0.000043	0.000053
2	8	0	-1.514073	0.327270	1.221556
3	8	0	-1.515849	-0.327021	-1.220469
4	6	0	0.448613	-1.310907	0.356185
5	6	0	0.448435	1.310529	-0.357354
6	6	0	1.780148	-0.755990	-0.146585
7	6	0	1.779745	0.756254	0.146634
8	1	0	0.111857	-2.214080	-0.144664
9	1	0	0.435001	-1.450763	1.435158
10	1	0	0.111357	2.214227	0.142329
11	1	0	0.435317	1.449050	-1.436507
12	1	0	1.870734	-0.924143	-1.221260
13	1	0	2.619606	-1.256921	0.334281
14	1	0	1.869288	0.924530	1.221376
15	1	0	2.619426	1.257479	-0.333535

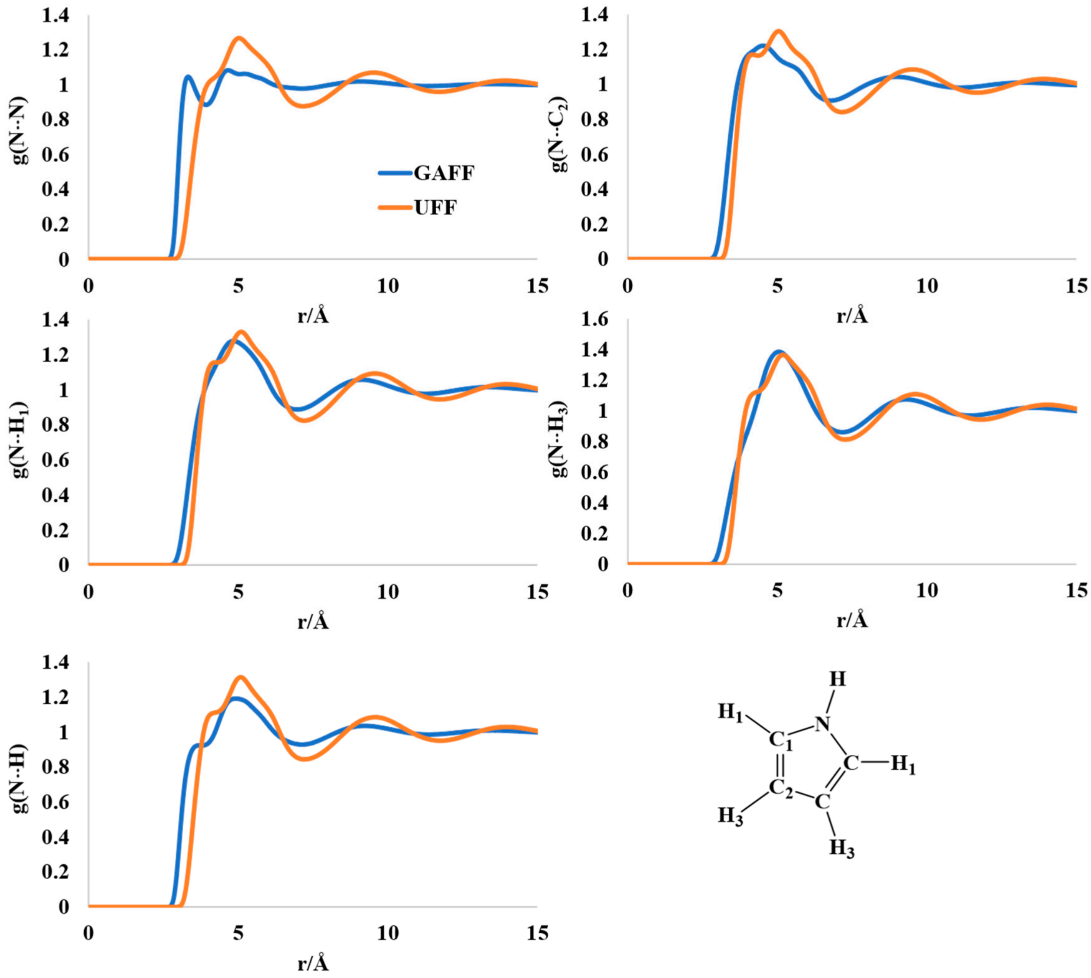

Figure S1. The GAFF and UFF forcefield computed pairwise distribution functions of liquid pyrrole via 3D-RISM-KH theory.

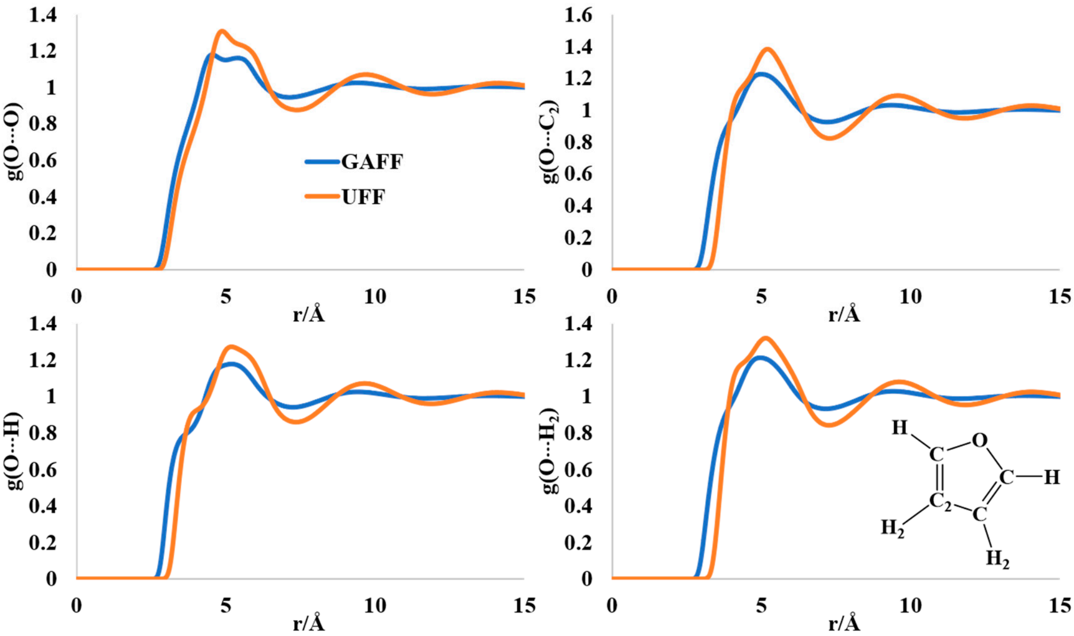

Figure S2. The GAFF and UFF forcefield computed pairwise distribution functions of liquid furan via 3D-RISM-KH theory.

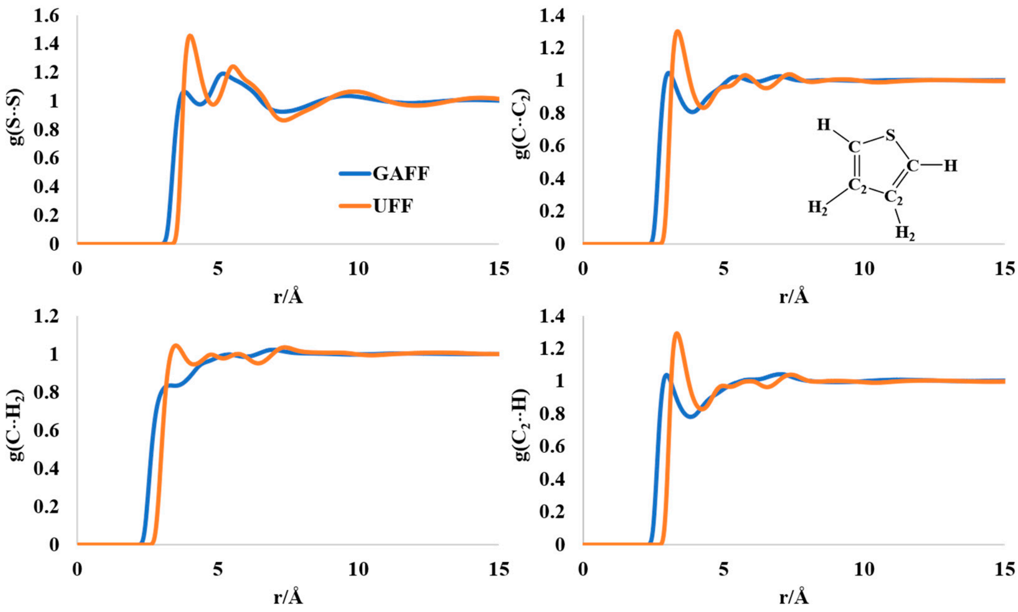

Figure S3. The GAFF and UFF forcefield computed pairwise distribution functions of liquid thiophene via 3D-RISM-KH theory.
